# Electromagnetic Fields and Human Endocrine System

**DOI:** 10.1100/tsw.2004.175

**Published:** 2004-10-20

**Authors:** Michal Karasek, Marta Woldanska-Okonska

**Affiliations:** ^1^ Department of Electron Microscopy, Chair of Pathomorphology, Medical University of Lodz, Poland; ^2^ Department of Endocrinology and Isotope Therapy, Medical University of Lodz; Polish Mother's Memorial Hospital – Research Institute, Lodz, Poland; ^3^ Chair of Rehabilitation, Swietokrzyska Academy, Branch of Piotrkow, Poland

**Keywords:** electromagnetic fields, endocrine system, hormones

## Abstract

Extremely low frequency electromagnetic fields (ELF EMF) are commonly present in daily life all over the world. Moreover, EMF are used in the physiotherapy of many diseases because of their beneficial effects. There is widespread public concern that EMF may have potential consequences for human health. Although experimental animal studies indicate that EMF may influence secretion of some hormones, the data on the effects of EMF on human endocrine system are scarce. Most of the results concentrate on influence of EMF on secretion of melatonin. In this review, the data on the influence of EMF on human endocrine system are briefly presented and discussed.

## INTRODUCTION

Extremely low frequency electromagnetic fields (ELF EMF) are commonly present in daily life all over the world. They are associated with the use of electric power applied in residential and occupational environments. Wherever electricity is generated, transmitted, or used, electric and magnetic fields are created due to the presence of motion of electric charges. They are emitted by power lines, electrical panels, transformers, and service wires, but also by such household appliances as televisions, electric blankets, hair driers, etc.[1]. Moreover, electromagnetic fields (EMF) are used in the physiotherapy of many diseases (e.g., low back pain syndrome, migraine and vasomotoric headaches, multiple sclerosis, degenerative processes of the bones and joints, rheumatoid arthritis) because of their beneficial effects (e.g., improvement of soft tissue regeneration processes, vasodilatory action, acceleration of bone adhesion formation, anti-inflammatory and analgesic action)[2,3].

There is widespread public concern that EMF may have potential consequences for human health[1,4,5], especially associated with increased risk for cancer and childhood leukemia[5].

Additionally some attention has also been paid to other possible health hazards, such as interference with cardiac pacemakers[6], Alzheimer's disease[7], and adverse pregnancy outcome[8].

Although there is still scientific controversy concerning that problem, it seems to be a growing consensus that human health hazard associated with exposure to EMF is either very small or restricted to small subgroups. A Working Group organized by the National Institute of Environmental Health Services concluded in a report published in 1998, on the basis of almost 900 publications, that: "None of the evidence for adverse health effects seen after exposure to ELF EMF achieved a degree of evidence exceeding 'inadequate' (for humans) or 'weak' (for experimental animals)"[1].

Studies on the effects of EMF in humans concentrate mainly on power line frequency fields or fields used in mobile phones. The influence of ELF EMF used in physiotherapy on the endocrine system was rarely examined.

Although experimental animal studies indicate that EMF may influence secretion of some hormones[9,10,11,12,13,14], the data on the effects of EMF on the human endocrine system are scarce. Most of the results concentrate on the influence of EMF on secretion of melatonin. In this review, the data on the influence of EMF on the human endocrine system are briefly presented and discussed.

## ELECTROMAGNETIC FIELDS AND MELATONIN

The data on the influence of EMF on melatonin secretion in humans are controversial. Although EMF-induced suppression on nocturnal melatonin secretion has been reported in occupational and residential studies[15,16,17,18,19,20], in the majority of laboratory-based exposure studies[21,22,23,24,25,26,27, 28,29,30,31,32,33,34,35,36,37,38,39], EMF did not exert a distinct influence on melatonin or 6-hydroxymelatonin concentrations[see 40] (Table 1). According to Karasek et al.[28,40], discrepancies in the results may depend on different experimental paradigms, including differences in certain characteristics of the applied magnetic fields, such as field induction, frequency, duration of exposure, timing of exposure, applied vector, etc.

## ELECTROMAGNETIC FIELDS AND PITUITARY HORMONES

No significant effects of EMF on the secretion of growth hormone and FSH have been found[32,39,41,42] (Table 2). Chronic application of 2.9 mT, 50 Hz EMF lowered the level of LH, whereas no changes were observed after chronic exposure to 25–80-µT, 200 Hz EMF[42] and acute exposure to 10 µT, 40 Hz (continuous or intermittent)[41] or to GSM-standard[32]. Woldanska-Okonska et al.[42] observed a decrease in prolactin concentrations following chronic application of EMF (25–80 µT, 200 Hz and 2.9 mT, 40 Hz, for 3 weeks) whereas acute exposure to 1 µT, 50 Hz[30] or 20 µT, 50 Hz did not influence the concentrations of this hormone[39].

## ELECTROMAGNETIC FIELDS AND PITUITARY – THYROID AXIS

Generally, EMF did not significantly influence hormones secreted by pituitary – thyroid axis[41,43] (Table 2). However, Woldanska-Okonska and Czernicki[43] observed differences in fT3 and fT4 levels after exposure to EMF of 2.9 mT, 40 Hz in comparison to 25–80 µT, 200 Hz 1 month following 3-weeks application. Moreover, among the studied patients, the authors have found the individuals especially sensitive to EMF in terms of secretion of TSH, fT3, and fT4.

**TABLE 1**
**Effects of Electromagnetic Fields on Melatonin or 6-Hydroxymelatonin Sulfate (6-OHMS) Concentrations in Humans[40, modified]**

| Exposure Parameters | Exposure Duration | Outcome | Ref. |
|---|---|---|---|
| | **Melatonin** | | |
| 150 mT (MRI) | 40.5 min | No effect | [21] |
| 1.5 T (MRI) | 60 min (01:00–02:00) | No effect | [22] |
| 10 µT, 50 Hz (continuous)<br>10 µT, 50 Hz (intermittent) | 9 h (23:00–08:00) | No effect | [23] |
| 1 µT, 60 Hz (intermittent)<br>20 µT, 60 Hz (continuous) | 8 h (23:00–07:00) | No effect (reduction in men with low baseline melatonin) | [24] |
| 20 µT, 50 Hz (continuous) | 8 h (23:00–07:00) | No effect | [25] |
| 20 µT, 50 Hz | 1.5–4 h (at night) | No effect (delayed rise) | [26] |
| 2.9 mT, 40 Hz | 3 weeks (16 min/day, 5 days/week) | Decrease | [27] |
| 25–80 µT, 200 Hz | 3 weeks (16 min/day, 5 days/week) | No effect | [28] |
| 1 µT, 50 Hz | 10 h (22:00–08:00) | No effect | [29] |
| 28.3 µT, 60 Hz | 8 h (23:00–07:00) | No effect | [30] |
| 100 µT, 50 Hz (continuous or intermittent) | 30 min (13:30–16:30) | No effect | [31] |
| GSM-standard: 900 MHz, pulsed with 217 Hz, pulse width of 577 µs | 8 h (23:00–07:00) | No effect | [32] |
| GSM-standard: 900 MHz, pulsed with 217 Hz, pulse width of 576 µs | 4 weeks (2 h/day, 5 days/week) | No effect | [33] |
| DCS-standard: 1800 MHz, pulsed with 217 Hz, pulse width of 576 µs | 4 weeks (2 h/day, 5 days/week) | No effect | [33] |
| GSM-standard: 900 MHz, pulsed with 217 Hz, pulse width of 577 µs | 20 randomly allotted 4-h sessions | No effect | [34] |
| GSM-standard: 900 MHz, pulsed with 217 Hz, pulse width of 576 µs | 60 min (19:00–20:00) | No effect | [35] |
| | **6-Hydroxymelatonin Sulfate** | | |
| 0.057 µT, 60 Hz<br>0.66 µT, 60 Hz<br>0.46 µT, 60 Hz | 4 weeks<br>4 weeks<br>7 weeks | No effect | [36] |
| 10 µT, 50 Hz (continuous)<br>10 µT, 50 Hz (intermittent) | 9 h (23:00–08:00) | No effect | [23] |
| 2–7 mT | 9 h (22:00–07:00) | No effect | [37] |
| 28.3 µT, 60 Hz | 8 h (23:00–07:00) | No effect | [30] |
| 100 µT, 50 Hz (continuous or intermittent) | 30 min (13:30–16:30) | No effect | [31] |
| 0.7–9.1 µT, 50 Hz | Overnight for 11 weeks | No effect | [38] |
| 20 µT, 50 Hz | 12 h (20:00–08:00) | No effect | [39] |

## ELECTROMAGNETIC FIELDS AND PITUITARY – ADRENAL AXIS

Electromagnetic fields did not influence secretion of ACTH[29]. In the majority of studies, no effect of EMF on cortisol secretion have been found[22,30,32,34,39,41] (Table 2). However, Woldanska-Okonska and Czernicki[44], studying cortisol concentration in four time points (06:00, 12:00, 16:00, and 24:00), observed different changes following chronic exposure to EMF of 2.9 mT, 40 Hz in comparison to 25–80 µT, 200 Hz. Exposure to EMF of 2.9 mT, 40 Hz resulted in decrease of cortisol levels at 16:00 h, whereas after exposure to EMF of 25–80 µT, 200 Hz, an increase in cortisol concentrations was found at 12:00 h.

**TABLE 2**
**Effects of Electromagnetic Fields on Concentrations of Various Hormones**

| Hormone | Exposure Parameters | Exposure Duration | Outcome | Ref. |
|---|---|---|---|---|
| FSH | 10 µT, 40 Hz (continuous) | 9 h (23:00–08:00) | No effect | [41] |
| | 10 µT, 40 Hz (intermittent) | 9 h (23:00–08:00) | No effect | [41] |
| | 2.9 mT, 40 Hz | 15 days, 20 min/day | No effect | [42] |
| | 25–80 µT, 200 Hz | 15 days, 20 min/day | No effect | [42] |
| LH | 10 µT, 40 Hz (continuous) | 9 h (23:00–08:00) | No effect | [41] |
| | 10 µT, 40 Hz (intermittent) | 9 h (23:00–08:00) | No effect | [41] |
| | 2.9 mT, 40 Hz | 15 days, 20 min/day | Decrease | [42] |
| | 25–80 µT, 200 Hz | 15 days, 20 min/day | No effect | [42] |
| | GSM-standard: 900 MHz, pulsed with 217 Hz, pulse width of 577 µs | 8 h (23:00–07:00) | No effect | [32] |
| GH | 20 µT, 50 Hz | 12 h (20:00–08:00) | No effect | [39] |
| | 1 µT, 50 Hz | 10 h (22:00–08:00) | No effect | [30] |
| | GSM-standard: 900 MHz, pulsed with 217 Hz, pulse width of 577 µs | 8 h (23:00–07:00) | No effect | [32] |
| Prolactin | 20 µT, 50 Hz | 12 h (20:00–08:00) | No effect | [39] |
| | 1 µT, 50 Hz | 10 h (22:00–08:00) | No effect | [30] |
| | 2.9 mT, 40 Hz | 15 days, 20 min/day | Decrease | [42] |
| | 25–80 µT, 200 Hz | 15 days, 20 min/day | Decrease | [42] |
| TSH | 10 µT, 40 Hz (continuous) | 9 h (23:00–08:00) | No effect | [41] |
| | 10 µT, 40 Hz (intermittent) | 9 h (23:00–08:00) | No effect | [41] |
| | 2.9 mT, 40 Hz | 15 days, 20 min/day | No effect | [43] |
| | 25–80 µT, 200 Hz | 15 days, 20 min/day | No effect | [43] |

## ELECTROMAGNETIC FIELDS AND GONADAL HORMONES

In men, chronic application of 2.9 mT, 40 Hz and 25–80 µT, 200 Hz EMF[42] or acute exposure to EMF of 1 µT, 50 Hz[30] did not affect testosterone concentrations. However, application of EMF of 25–80 µT, 200 Hz lowered levels of estradiol, whereas EMF of 2.9 mT, 40 Hz have no effect in men[42].

## CONCLUDING REMARKS

In general, it seems that EMF exert no or very subtle effects on the endocrine system. Small differences reported in various studies may depend on different characteristics of applied magnetic fields and different experimental paradigm.

## ACKNOWLEDGMENTS

The work by the authors and this review were supported by a grant from Medical University of Lodz, No. 503-129-2.
